# Enzymatically Regulated Peptide Pairing and Catalysis for the Bioanalysis of Extracellular Prometastatic Activities of Functionally Linked Enzymes

**DOI:** 10.1038/srep25362

**Published:** 2016-05-03

**Authors:** Hao Li, Yue Huang, Yue Yu, Tianqi Li, Genxi Li, Jun-ichi Anzai

**Affiliations:** 1 State Key Laboratory of Pharmaceutical Biotechnology and Collaborative Innovation Center of Chemistry for Life Sciences, Department of Biochemistry, Nanjing University, Nanjing 210093, China; 2 Nanjing Drum Tower Hospital, The Affiliated Hospital of Nanjing University Medical School, Nanjing 210008, China; 3 Laboratory of Biosensing Technology, School of Life Sciences, Shanghai University, Shanghai 200444, China; 4 Graduate School of Pharmaceutical Sciences, Tohoku University, Aramaki, Aoba-ku, Sendai 980-8578, Japan

## Abstract

Diseases such as cancer arise from systematical reconfiguration of interactions of exceedingly large numbers of proteins in cell signaling. The study of such complicated molecular mechanisms requires multiplexed detection of the inter-connected activities of several proteins in a disease-associated context. However, the existing methods are generally not well-equipped for this kind of application. Here a method for analyzing functionally linked protein activities is developed based on enzyme controlled pairing between complementary peptide helix strands, which simultaneously enables elaborate regulation of catalytic activity of the paired peptides. This method has been used to detect three different types of protein modification enzymes that participate in the modification of extracellular matrix and the formation of invasion front in tumour. In detecting breast cancer tissue samples using this method, up-regulated activity can be observed for two of the assessed enzymes, while the third enzyme is found to have a subtle fluctuation of activity. These results may point to the application of this method in evaluating prometastatic activities of proteins in tumour.

The biochemical microenvironment is maintained in dynamic balance by an orchestrated interplay of proteins and other components whose bioactivities are constantly regulated. This network of protein interactions can be disrupted by pathological development such as cancer^1^,^2^,^3^, resulting in characteristic changes of protein interactions significant for understanding and controlling carcinogenesis and other pathological development. Consequently, analytical tools are needed to survey the inter-connected activities of multiple proteins in a disease-associated context. However, for this kind of application, the existing methods are restricted in two respects. First, the targeting probes are not designed for the detection of protein activity. For example, immunoassays employ antibodies which, as a matter of fact, are raised against epitopes that may or may not be present on the surface of protein under native conditions. So antibodies can always recognize the target proteins regardless of their activity, or even recognize only the exposed epitopes of denatured proteins, as in the case of most western blotting protocols using denatured protein samples. As for aptasensors, aptamers targeting bioactive proteins are usually screened against the active center, as in the case of most protease-targeting aptamers, abolishing the possibility of aptamer-based protein activity assay. Second and more importantly, although there have been multiplexed protein assays, the targets are usually biomarkers independently developed, so their activities often have no much connections with each other, given the exceedingly large number of deregulated proteins in disease such as cancer. These multiplexed assays can only monitor the advancing of disease, but can hardly lend any help to promote the current understanding of the molecular mechanism. It thus still remains challenging to come up with an activity assay for analyzing the dynamics of several functionally inter-linked proteins.

In this work, a method is established for several correlated protein modification enzymes involved in the formation of invasion front during metastatic development. This design is inspired by the pattern of protein interactions in the cell. The network of protein interaction contains many converging points where the same protein is acted upon by many different ligands. Accordingly, a peptide probe can be constructed, whose conformation and catalytic activity can be regulated by the activity of the several functionally related enzymes.

Peptides are the structural elements of protein, so, to some extent, peptides possess the chemical and conformational diversity of proteins that enable interactions with a variety of partners^4^,^5^,^6^,^7^,^8^,^9^,^10^,^11^,^12^,^13^,^14^. Biosensing devices^15^,^16^,^17^,^18^,^19^ based on the conformational switching of peptides can be constructed from peptides via their interactions with the bioactive target proteins. But the challenge in reducing such ideas to practice lies in the subtle conformation of peptide, which, although enabling finely tuned interactions with proteins, lacks the definiteness in DNA conformation that constitutes the sound basis of an “all-or-nothing” biosensing switch^20^,^21^,^22^,^23^,^24^. To build distinct conformational change into peptide biosensing devices, we propose a new kind of design strategy by employing peptide cyclization to control the formation of coiled-coil motif (Fig. 1a). Coiled-coil is a super helix formed by two complementary strands of alpha-helix twisted around each other^25^. The pairing of coiled-coil peptide enables many interesting biosensing applications^26^,^27^,^28^, while the control of pairing by covalent cyclization of the peptide has not been attempted before. The pairing of the two strands might be prevented by main-chain cyclization over a considerable length of the strand^29^. To convert such covalently controlled conformational changes into signal readout, a cupric ion binding motif with catalytic activity is split on the two complementary strands^30^, so that formation/dissociation of the Cu (II) binding motif, in consequence the acquiring or losing of catalytic ability, always accompany the pairing/separating of the two complementary strands. In turn, this pairing/separating of the complementary strands can be realized by loop-formation or loop-opening of the strand controlled by various types of protein modification enzymes such as proteases and trans-peptidase, as well as enzymes that modify the side chain groups of amino acids^31^,^32^,^33^,^34^. In this way, enzyme activities can control both peptide pairing and peptide catalysis, enabling an assay of three different types of enzymes. These three types of extracellular enzymes are frequently observed with simultaneous elevation of activity in metastasis^35^,^36^,^37^, and the simultaneous assay of the activity of these three inter-connected protein modification enzymes may unveil the interaction between these enzymes and other components of the tumour microenvironment^38^,^39^,^40^,^41^, so as to improve the current understanding of metastatic cancer.

## Results

### Design principle

**Figure 1:**
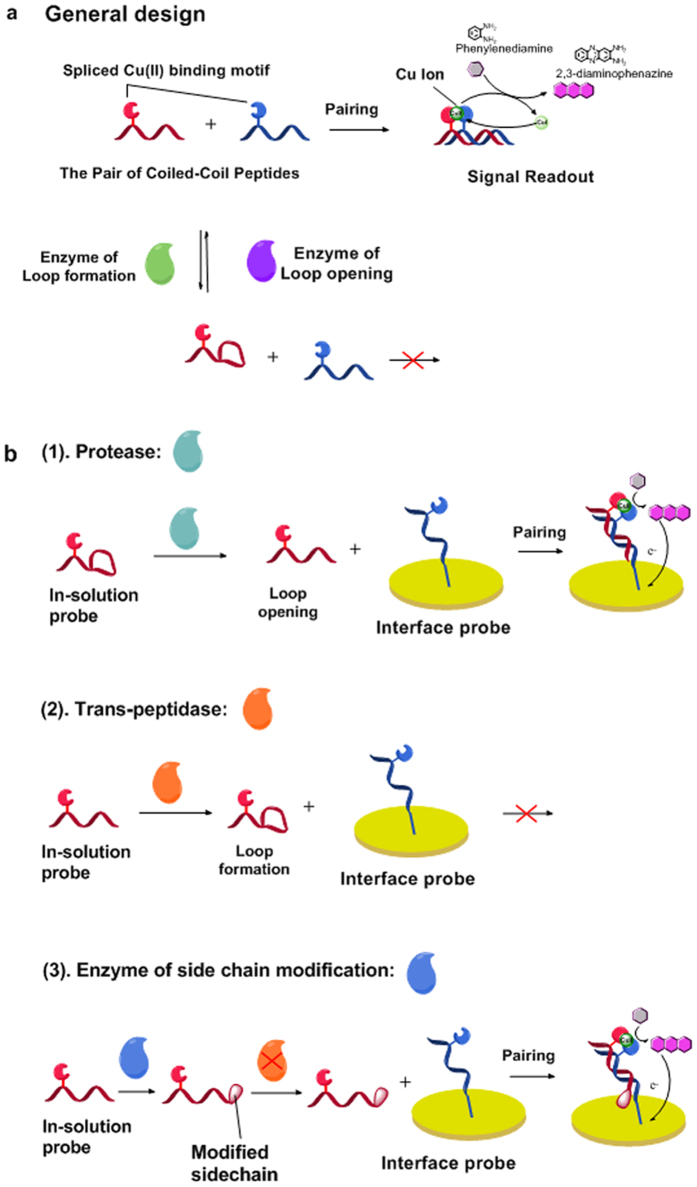
Design of the proposed peptide-based assay. (**a**) General design of the peptide probes for enzyme biosensing: activity of the target enzymes controls the loop formation/opening on one of the pair of peptides, and this in turn controls the pairing of the two peptides. The pairing of the peptides leads to signal readout by enabling Cu ion catalyzed oxidation of inactive *ο*-phenylenediamine to electro-active 2,3-diaminophenazine. (**b**) Specialized applications of the probes in detecting three different types of protein modification enzymes of the remodeling of tumour microenvironment.

The general design proposed in this work, illustrated in Scheme 1a, has been tailored and modified to assay the activity of three types of protein modification enzymes involved in tumour invasion (Fig. 1b). First, in detecting protease, a looped in-solution probe is adopted, while a linear alpha-helix is immobilized on electrode surface as capturing probe. Protease cleavage will free the in-solution probe from the loop to pair with the capturing probe and to form the coiled-coil, giving rise to catalytically amplified signal readout positively correlated with protease abundance (Fig. 1b(1)). Second, in detecting trans-peptidase, the two short sequences recognized by the target enzyme are respectively elongated at one end and branched out along the chain of the linear in-solution probe, so that the enzyme activity creates a loop that inhibits coiled-coil formation, resulting in signal reversely proportional to enzyme concentration (Fig. 1b(2)). Third, in detecting the enzymes responsible for side chain modification, trans-peptidase is employed as a tool enzyme. Activity of the target enzyme results in modification of target amino acids in the trans-peptidase recognition sequence, blocking the trans-peptidase reaction, thereby preserving the linear form of the in-solution probe for it to form the coil with the interface probe (Fig. 1b(3)), a signal response proportional to enzyme abundance can thus be achieved.

To better realize the above design, we have modified the original coiled-coil sequence^42^, in light of some considerations concerning the proper conformation of coiled-coil. First, only part of the helix can be constrained in the loop, since the cyclization of the whole helix carries great entropy penalty, detrimental to the reliability of the working principle. Second, all the sequence modifications, including the split cupric ion binding motif, should orient away from the polyleucine hydrophobic patch along the helix, to avoid interfering with coiled-coil formation. Third, the two strands of coiled-coil are to some extent comparable with paired DNA strands, meaning that they are complementary rather than equivalent, so the pairing between linear in-solution probes is unlikely to happen.

**Figure 2:**
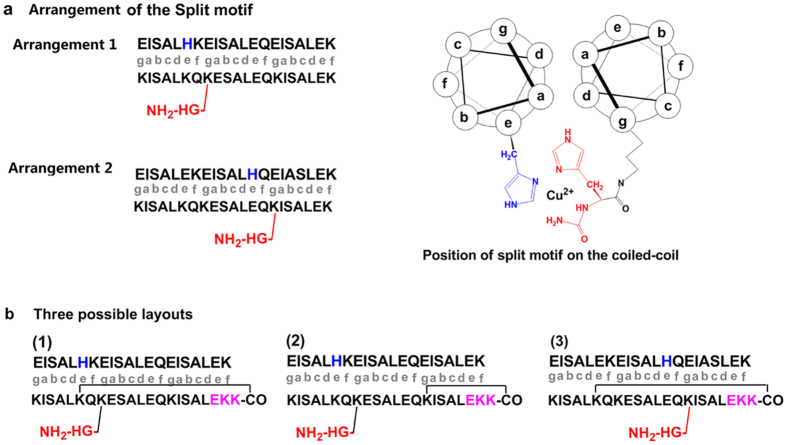
Detailed design of the peptide sequences. (**a**) Two viable arrangements of the split cupric ion binding motif on the peptide probe. Left: sequences of the pair of peptide probes, and the modifications are marked with color: the blue and red colored groups are the two halves of the spliced Cu ion binding motif. Lower-case letters in gray represent positions of amino acids as well as the modifications on the helical-wheel depiction of the pair of peptide probes (at right). (**b**) Three possible layouts of plasmin probes based on the two designs in (**a**). The side chain cross-linked amino acids are linked with lines. The violet “EKK” represents the cleavage sequence of plasmin, and the other colored letters are of the same meaning as in (**a**).

Although the two “anti-parallel” strands of coiled-coil contains many glutamic acid and lysine residues potentially available for branching and cyclization, only few of them are suitable for forming the split cupric ion-binding motif, which is designed to be a histidine substitution on one strand, with a corresponding N-Gly-His dimeric branch on the other. This is because the side chain of every 8^th^ amino acid projects to the same direction. Therefore, to orient the two split halves to the same direction and to place them in close vicinity, only two pairs of sites are available (Fig. 2a) on the two complementary strands. On the other hand, the cyclization can also be designed to take place at two different sites along the strands. And to simplify the design, only one of the two strands is designed to be cyclizable. So, three arrangements have been designed and obtained (Fig. 2b).

### Validation of design principle

The above designs have been first tested in protease detection. Isothermal titration calorimetry (ITC) is employed to monitor the interaction of the looped probe with its counterpart, before and after protease cleavage, as well as the function of the split motif when pieced together via coiled-coil formation (Supplementary Fig. S1~3). Layout 1 with closely situated split motif and cyclization site shows hampered Cu (II)-binding ability (Supplementary Fig. S3a). As for layout 2, the smaller loop close to one end cannot effectively block coiled-coil formation in the absence of protease activity (Supplementary Fig. S1b). On the contrary, the lactam bridge so formed may even facilitate the coiled-coil formation in the absence of protease activity, leaving only layout 3 as the more rational design (Fig. 2b(3)). In this design, the looped probe has a N-Gly-His branch enclosed by the loop. So this probe can be employed as the in-solution probe, and its counterpart, the linear strand, can be immobilized on the electrode surface as the interface probe. Since this basic probe design can serves as the prototype for the following assays of all the three enzymes, the common final step in the three, or the forming of coiled-coil and binding of cupric ion, is first investigated. As described above in Fig. 1a, in this final step, the cupric ion complexed by the paired probes can generate amplified signal readout via catalysis of *ο*-phenylenediamine (OPD) oxidation to 2,3-diaminophenazine (DAP). Using this signal readout, the condition for coiled-coil formation on the electrode surface has also been optimized. As is shown in Supplementary Fig. S4, this interaction is found to complete in 60 min.

### Detection of enzymes

**Figure 3:**
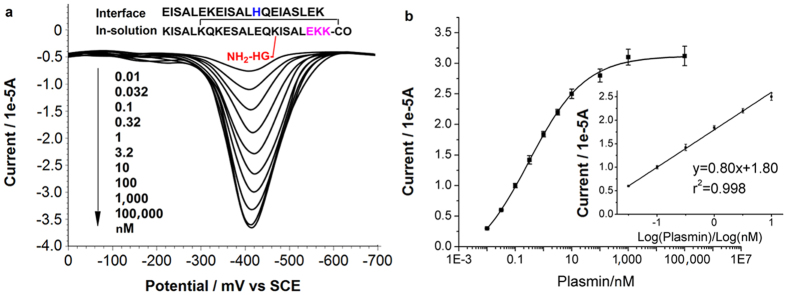
Quantitative analysis of active plasmin using the peptide probe. (**a**) Square wave voltammograms (SWVs) of DAP showing the gradual increase of signal response with plasmin concentration. The sequence of the two probes forming the switch has also been presented. In the detection procedure, the in-solution probe is first acted upon by the target enzyme, and then the reaction mixture is incubated with the interface probe-modified working electrode. The electrode is subsequently transferred into the solution of signal amplification, and the electro-activity of DAP generated in this solution is finally recorded. (**b**) Peak currents in (**a**) plotted as a function of plasmin concentration. Inset is the linear range and the corresponding formula obtained via regression analysis. The error bars represent standard deviation from average (n = 3).

**Figure 4:**
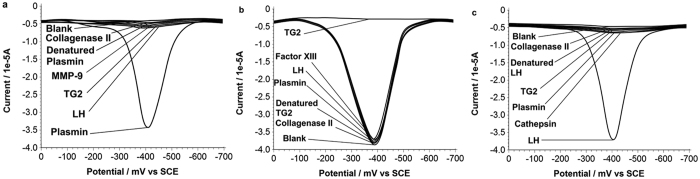
SWVs of DAP showing the specificity of the assays for the three enzymes, (**a**) plasmin assay, (**b**) TG2 assay, (**c**) LH assay. The signal readout is obtained as same as in Fig. 3. All control species for (**a**) are of 10 nM, for (**b**), 1.6 μM, for (**c**), 0.32 μM.

Employing the above probe design, protease detection is first demonstrated using plasmin, a serine protease actively participating in the breakdown of extracellular matrix (ECM) for tumour invasion^43^. The assay is conducted according to the 1^st^ design variant in Fig. 1b, while the specific sequence of the corresponding peptide probe has been shown in Fig. 3a. The effectiveness of the peptide probe has also been examined by the detection of control species (Fig. 4a). Experimental results reveal that the blank control has a response of background level, indicating that the main-chain cyclization of the in-solution probe can block its interaction with the interface probe, in the absence of enzyme cleavage, consistent with the above ITC results (Supplementary Fig. S1, S2). It can also be concluded that other ECM enzymes cannot recognize the plasmin substrate contained in the probe, resulting in background response. So the specificity of the plasmin assay is satisfactory, and in the detection of clinical tissue samples, the other ECM enzymes in the sample will not lead to serious interference. Plasmin cleavage time has been optimized as well (Supplementary Fig. S5), and it is found that the cleavage can complete in less than 30 min. Further studies reveal that the response of catalytically generated DAP increases proportionally to the logarithm of protease concentration from 0.032 nM–10 nM (Fig. 3), with a limit of detection of 0.002 nM, defined at signal-to-noise ratio of 3:1. The average standard deviation of all repetitive measurements is within 5%, showing an acceptable reproducibility.

**Figure 5:**
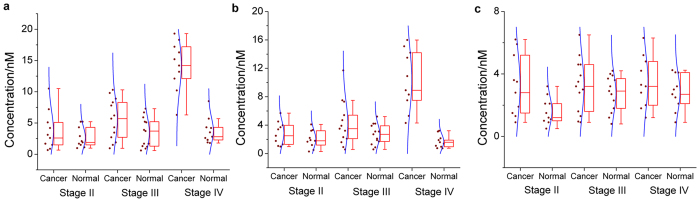
Box chart to show the distribution of the detected concentration of active (**a**) TG2, (**b**) LH and (**c**) plasmin, in patient with breast cancer of different stages, The signal readout is obtained as same as in Fig. 3. For each sample a corresponding result from the paracancerous normal tissue has been included as a control. Each box includes the maximum, minimum, mean, 1st and 99th percentile marked on the graph, in addition to the 25th, median, and 75th percentiles. The raw data is included as a column scatter plot to the left of each box. A curve corresponding to normal distribution is also displayed on top of the scatter plot.

Using the 2^nd^ design variant shown in Fig. 1b and the corresponding pair of peptide probes as shown in Fig. 5a, tissue transglutaminase 2 (TG2), a trans-peptidase that can crosslink and thereby remodel the fabrication of ECM^44^,^45^,^46^, can be detected in the range of 0.1 nM–1.6 μM (Supplementary Fig. S6), under the optimized condition (Supplementary Fig. S7). The limit of detection is 0.027 nM. The specificity of this assay is also satisfactory, for instance, the other two ECM enzymes studied in this work cannot interfere with the detection (Fig. 4b).

Design variant 3, shown in Fig. 1b, is employed to detect lysyl hydroxylase (LH), recently found to be strongly connected with metastatic cancer^39^,^47^. Up regulated LH can result in re-location of tumour cells towards front edge of invasion. Using the specifically designed probe, the sequence of which is shown in Supplementary Fig. S8a, LH can be detected in the range of 0.1 nM–0.32 μM (Supplementary Fig. S8b), under the optimized condition (Supplementary Fig. S9). The limit of detection is 0.036 nM. The specificity of this assay is also satisfactory (Fig. 4c).

### Detection of clinical samples

To observe the effect of these three ECM remodeling enzymes in promoting invasion, a bioanalysis of tissue activity of protein modification enzymes in 33 cases of breast cancer victims has been conducted using the proposed method. The samples are sub-grouped according to their stages defined by pathological examination. (Fig. 5). Pathologically more advanced cases, with higher stage, less differentiation, even with initial metastasis to sentinel lymph nodes, are connected with evidently elevated TG2 and LH activity (Fig. 5a,b, respectively). Consistent with these observations, previous researches confirm that multiple hypoxia-induced cell signaling finally lead to TG2 and LH controlled remodeling of ECM frameworks and ultimately triggering critical events in metastasis, such as epithelial-mythenchymel transition, angiogenesis and invasion^2^,^48^,^49^. On the contrary, plasmin activity displays no apparent correlation with the advancing of cancer (Fig. 5c). Correspondingly, a body of findings, gathered over decades, has suggested the activation of plasmin by urikinase-type plasminogen activator as a hallmark of metastatic and therapy-resistant breast cancer, although the underlying mechanisms connecting plasmin activity to metastatic breast cancer might be a little subtle^50^.

## Discussion

In this work, we have designed a peptide conformational probe to detect three different kinds of enzymes of tumour microenvironment modifications. The peptide probe has a modified coiled-coil structure, the formation of which can be covalently controlled by the activity of the target enzymes. Meanwhile, this covalently controlled conformational rearrangement can result in the formation of a cupric ion binding motif that can catalytically convert the above molecular recognition and structural reconfiguration into amplified signal readout. Using this method to analyze clinical samples of breast cancer, two enzymes are observed with evidently higher activity for metastatic cases examined, while one enzyme has a subtly fluctuated abundance, in relative to the stage of cancer. Based on these results, it might be proper to conclude that this peptide conformational probe might possess the potential for more extensive use in investigating tumour-promoting activities of proteins, such as studying the function of proteases in the formation of invasion front during epithelial-mesenchymal transition.

## Method

### Chemicals and Reagents

Peptide probes (their sequences as shown in Figs 2b and 3 and Supplementary Fig. S6, S8) were manufactured by Shanghai Science Peptide as lyophilized powder, purity >90%, specifically, the in-solution probes of plasmin had a peptide-bond between the C-terminal carboxyl and the side chain amine of a lysine residual in the sequence. For the interface probes, 11-mercaptoundecanol was incorporated into their sequences for surface immobilization of these probes. Human recombinant palsmin was from Milipore. Recombinant transglutaminase 2 (TG2) was provided by R&D Systems. Lysyl hydroxylase (LH) was purchased from Bioabb. Analytical-grade was warranted for all of the other reagents used. Powder of the surface-immobilized probes were dissolved with 10 mM phosphate buffer solution (PBS) (pH 7.4) to prepare 5 μM stock solutions, while powder of the in-solution probes were dissolved to 100 μM with the corresponding buffers of the target enzymes as listed below. Powder of all the enzymes were dissolved with proper solution to prepare standard samples. The buffer adopted for plasmin was 10 mM PBS, pH 7.4; for TG2: 10 mM Tris-HCl, 0.14 M NaCl, 30 mM CaCl_2_, pH 8.3; for LH: 50 mM Tris-HCl, 0.08 mM FeSO_4_, pH 7.8. Redistilled water for preparation of all the solutions was produced with a Milli-Q purification system, the resistance of 18 MΩ·cm was achieved to guarantee the purity. Biopsy samples of patients of breast cancer were obtained from Nanjing Drum Tower Hospital, the Affiliated Hospital of Nanjing University, in accordance with the approved guidelines. All experimental protocols were approved by elected consent of the Ethical Committee of Nanjing Drum Tower Hospital, and consent had also been obtained from all subjects. Upon receiving the samples, they were directly sliced on ice to 1 mm^3^, followed by digestion with type II collagenase at 37 °C for 30 min. The resulted supernatant was centrifugated at 800 rpm for 5 min, the cell free supernatant thus obtained was 10× diluted and immediately brought to detection procedures. The methods were carried out in accordance with the approved guidelines.

Statement: the samples were obtained in accordance with the approve guidelines, and informed consent had also been obtained from all subjects. All experimental protocols were approved by elected consent of the Ethical Committee of Nanjing Drum Tower Hospital.

### Electrode Treatment and Modification

These steps were essentially the same as previously reported^17^. Briefly, the electrode was reacted with 5 μM peptide in 10 mM PBS (pH 7.4) at 4 °C for 16 h, followed by being dipped in 1 μM 9-mercaptononanol for 3 h.

### Enzyme Activity Assay

Firstly, standard or clinical samples were mixed at a volume ratio 1:10 with the in-solution probes and incubated at 37 °C for proper time. Then, the reaction mixture was incubated with the peptide modified electrodes at ambient temperature (25 °C) for 1 h, in the presence of 100 μM CuCl_2_. After that, the electrode was gently rinsed with double-distilled water. To generate readout signal, the electrode was dipped in a 1 mM HCl solution (1 mL) containing 0.1 mg/ml *ο*-phenylenediamine (OPD). The solution was then placed in 50 °C water bath for 30 min. After being cooled to room temperature, this solution was buffered with 4 mL 10 mM PBS pH 7.4. Signal response of the generated 2,3-diaminophenazine (DPA) was then recorded in the buffered reaction solution.

### Experimental Measurements

These steps were essentially the same as previously reported^18^. Briefly, isothermal titration calorimetry (ITC) measurements were conducted using a MicroCal ITC200 System (GE healthcare life sciences). The titration was conducted at 25 °C. The titration schedule consisted of 38 consecutive injections of 1 μL with at least a 120 s interval between injections. Heats of dilution, measured by titrating beyond saturation, were subtracted from each data set. All solutions were degassed prior to titration. The data were analyzed using Origin 7.0 software. Electrochemical measurements were carried out on a CHI660D Potentiostat (CH Instruments) with a conventional three-electrode system: the electrode immobilized with peptide as the working electrode, a saturated calomel electrode (SCE) as the reference electrode and a platinum wire as the counter electrode. Square wave voltammograms (SWVs) were recorded in 10 mM PBS, pH 7.4, which was deoxygenated by purging with nitrogen gas and maintained under this inert atmosphere during the electrochemical measurements.

## Additional Information

**How to cite this article**: Li, H. *et al*. Enzymatically Regulated Peptide Pairing and Catalysis for the Bioanalysis of Extracellular Prometastatic Activities of Functionally Linked Enzymes. *Sci. Rep*. **6**, 25362; doi: 10.1038/srep25362 (2016).

## Supplementary Material

Supplementary Information
