# Supplementary material for: Enzymatically Regulated Peptide Pairing and Catalysis for the Bioanalysis of Extracellular Prometastatic Activities of Functionally Linked Enzymes
Source: Sci Rep. 2016 May 3;6:25362. doi: 10.1038/srep25362 (PMC4853721; doi:10.1038/srep25362)
Supplement: Supplementary Information [file srep25362-s1.pdf]

Supporting Information for

Enzymatically Regulated Peptide Pairing and Catalysis for the Bioanalysis of Extracellular Prometastatic Activities of Functionally Linked Enzymes

Hao Li[1], Yue Huang[1] Yue Yu[2], Tianqi Li[1], Genxi Li[1, 3,*], Jun-ichi Anzai[4]

1. State Key Laboratory of Pharmaceutical Biotechnology and Collaborative Innovation Center of Chemistry for Life Sciences, Department of Biochemistry, Nanjing University, Nanjing 210093, China.

2. Nanjing Drum Tower Hospital, The Affiliated Hospital of Nanjing University Medical School, Nanjing 210008, China.

3. Laboratory of Biosensing Technology, School of Life Sciences, Shanghai University, Shanghai 200444, China.

4. Graduate School of Pharmaceutical Sciences, Tohoku University, Aramaki, Aoba-ku, Sendai 980-8578, Japan.

*Corresponding author at State Key Laboratory of Pharmaceutical Biotechnology, Department of Biochemistry, Nanjing University, Nanjing 210093, China. Fax: +86 25 83592510. E-mail address: genxili@nju.edu.cn.

List of contents:

**Suppl. Figures**		
**1**	**Isothermal calorimetric validation of design**	**pS2~S4**
**2**	**Optimization of various experimental conditions**	**pS5~S10**

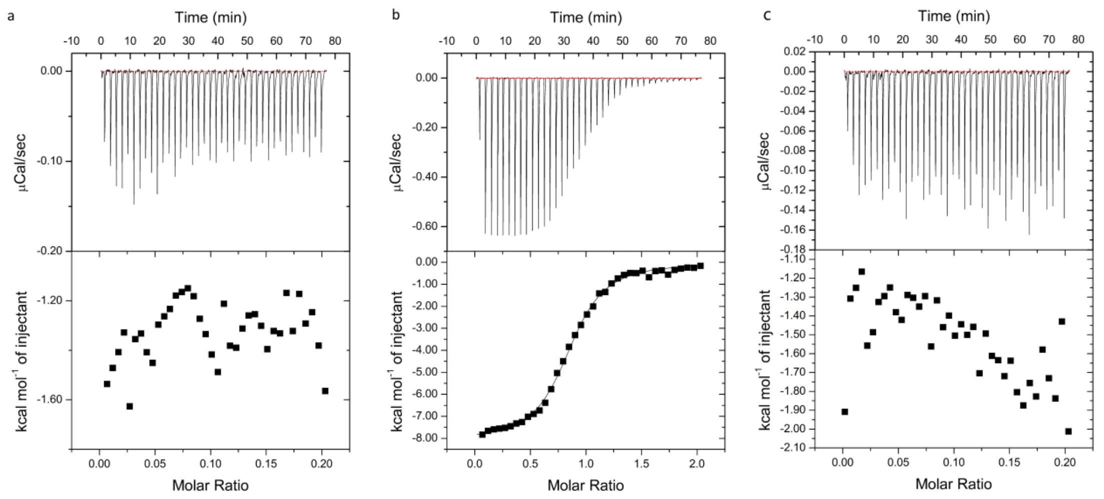

Figure S1. Isothermal calorimetry (ITC) data obtained by titrating 1 mM of the three types of “in-solution” peptide probes of plasmin shown in Fig. 2b into 0.1 mM of their counterpart “interface” probes, in the absence of plasmin cleavage. The solution adopted for both the “in-solution” and “interface” probes are in accord with that used in the activity assay. The top row displays the raw data of power versus time. The bottom row is the corresponding data by integrating enthalpy values versus the molar ratio of titrant: titrand. These data are fit using Origin 7.0 software, the resulted fitting curve is also shown in the lower row of (b).

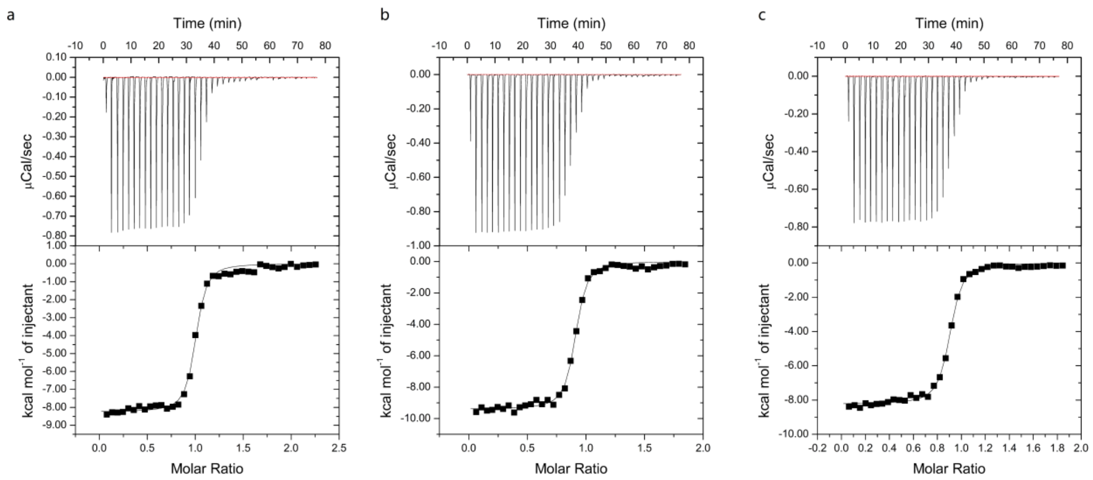

Figure S2. Results of the same experiments conducted after plasmin cleavage, namely, the in-solution probes have been incubated with excessive amount of plasmin at first, before been used for titration. There is no apparent interaction between plasmin and the “interface” probes (data not shown). The meaning of all the data points and curves are the same as Figure S1.

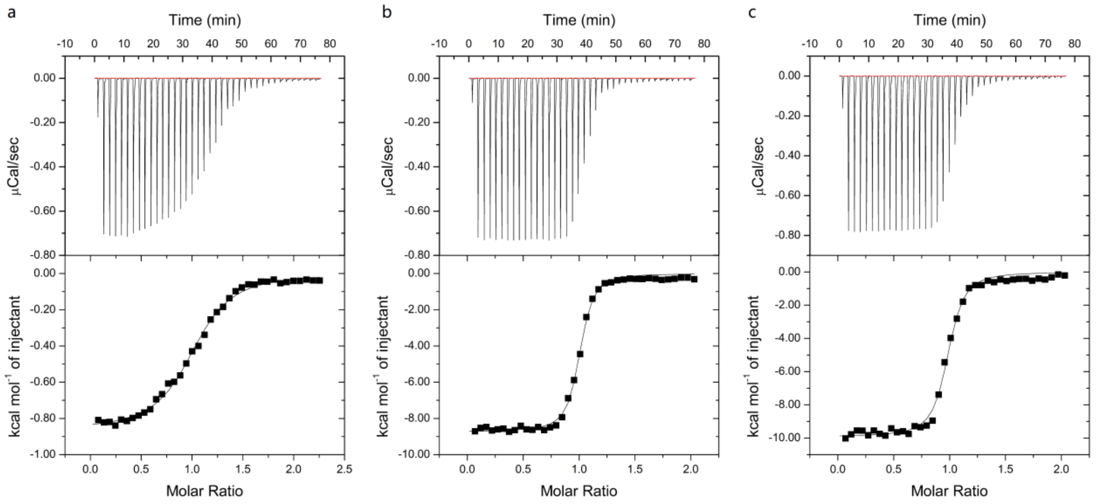

Figure S3. a - c are the ITC data obtained by titrating 1 mM cupric ion into the three types of paired peptide probes for plasmin as shown in Fig. 2b. In all three measurements, 0.1 mM of the “in solution” probes have been incubated with 0.1 mM “interface” probes at first, to form the paired probe. The solution adopted for both the “in-solution” and “interface” probes are in accord with that used in the activity assay. The meaning of all the data points and curves are the same as the former two figures.

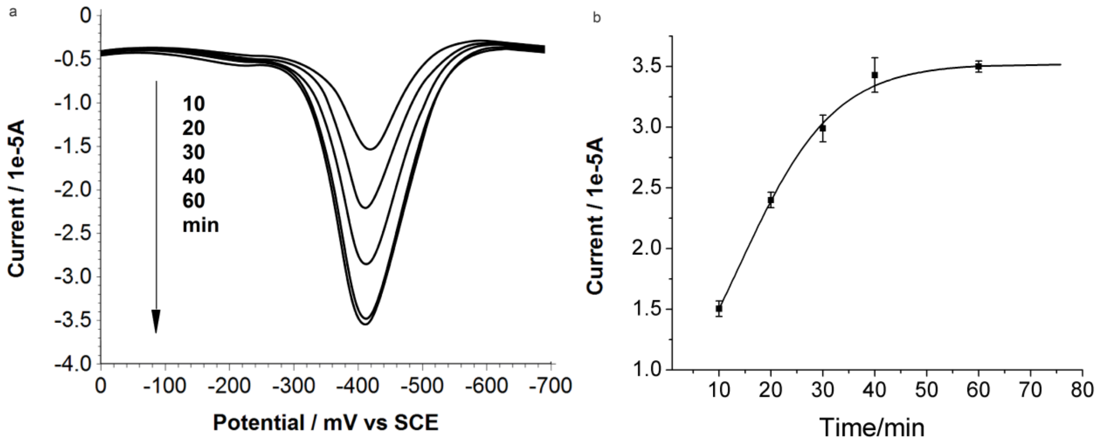

Figure S4. Optimization of the incubation time for probe binding. (a) Square wave voltammograms (SWVs) of 2,3-diaminophenazine (DAP) showing the effect on final signal readout, of incubation time for the interaction between the in-solution probe and the interface probe of layout 3 in Scheme 2b, in the presence of 100 μM cupric ion. The interface-probe modified electrode has been sequentially incubated with the in-solution probe, then with cupric ion for catalytic generation of DAP. (b) Peak currents in (a) plotted as the function of interaction time (Error bars mark the standard deviation, n=3).

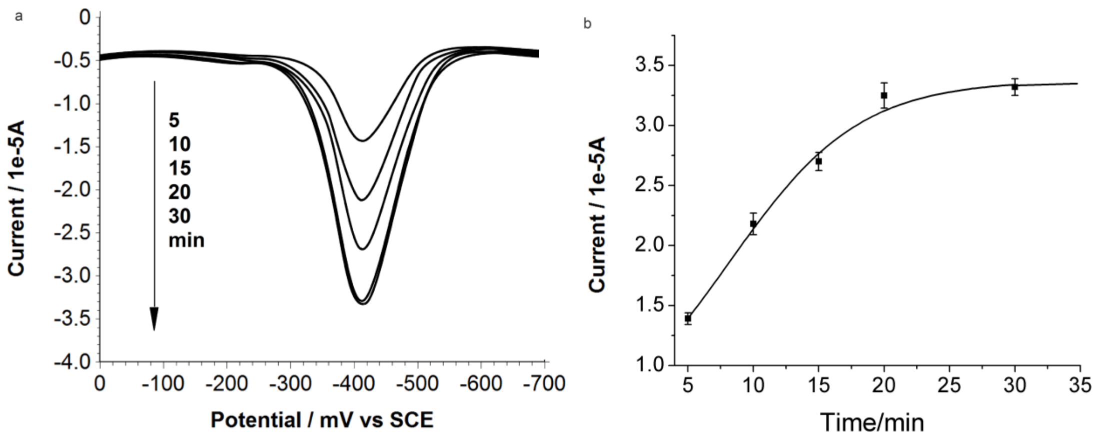

Figure S5. Optimization of the incubation time for plasmin cleavage. (a) SWVs of DAP showing the influence of the time of plasmin cleavage on the final signal readout. The peptide-modified electrode has undergone the full procedure of detection as described in the experimental section (Target concentration, 10 nM). Particularly, a series of different incubation time for plasmin cleavage has been adopted. (b) Peak currents in (a) plotted as the function of cleavage time (Error bars mark the standard deviation, n=3).

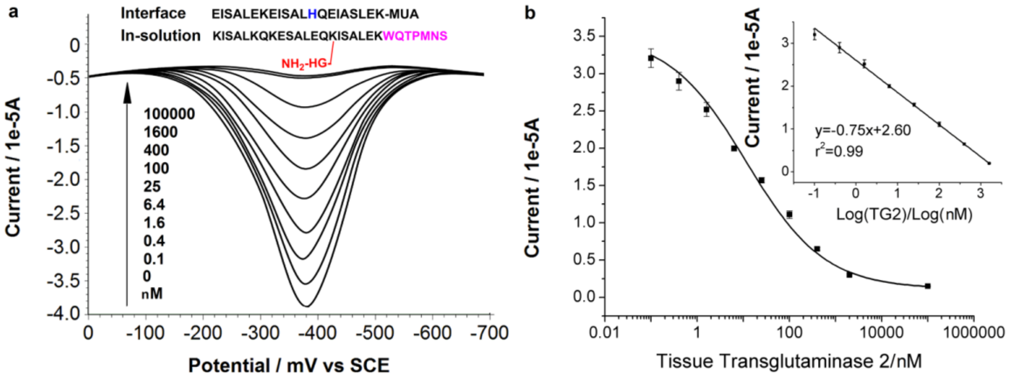

Figure S6. Quantitative results of TG2 activity assay. (a) Square wave voltammograms (SWVs) of DAP showing the gradual increase of signal response with plasmin concentration. The sequence of the two probes forming the switch has also been presented. In the detection procedure, the in-solution probe is first acted upon by the target enzyme, and then the reaction mixture is incubated with the interface probe-modified working electrode. The electrode is subsequently transferred into the solution of signal amplification, the electro-activity of DAP generated in this solution is finally recorded. (b) Peak currents in (a) plotted as a function of plasmin concentration. Inset is the linear range and the corresponding formula obtained via regression analysis. The error bars represent standard deviation from average (n=3)..

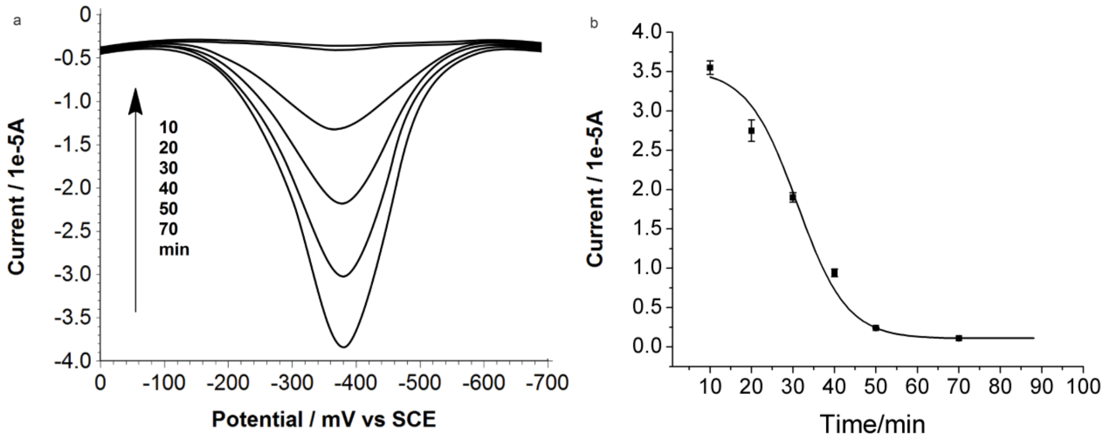

Figure S7. Optimization of the time of TG2-catalyzed reaction. (a) SWVs of DAP. The peptide-modified electrode has undergone the full procedure of detection as described in the experimental section (Target concentration, 1.6 μM). Particularly, a series of different incubation time for TG2-catalyzed reaction has been adopted. b) Peak currents in (a) plotted as the function of reaction time (Error bars mark the standard deviation, n=3).

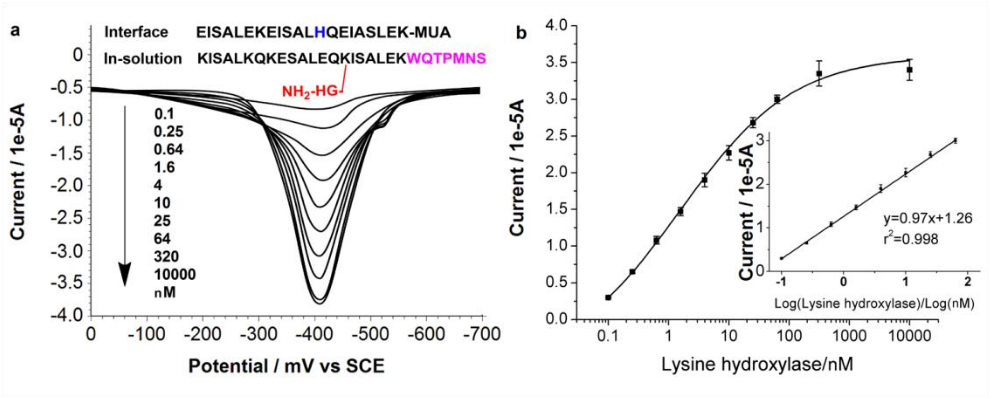

Figure S8. Quantitative results of LH activity assay. The meaning of all the data points and curves are the same as Figure S6.

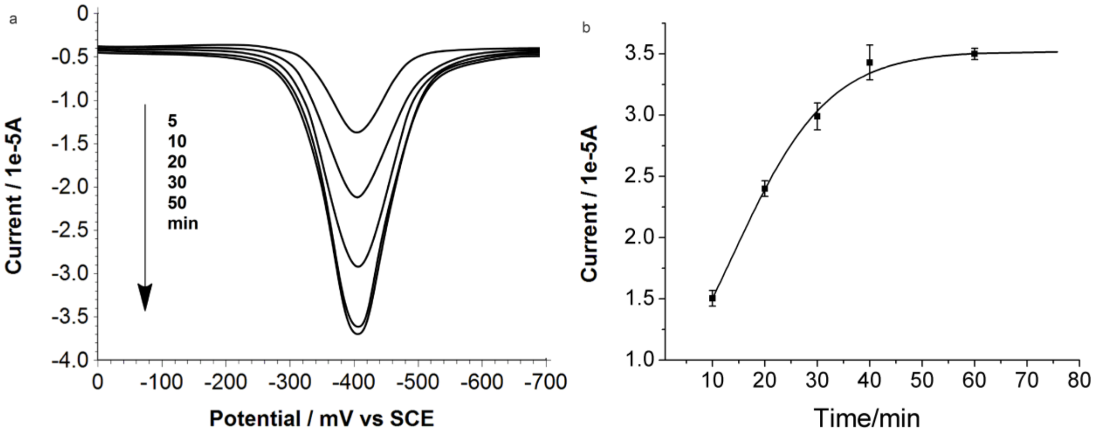

Figure S9. Optimization of the time of TG2-catalyzed reaction. (a) SWVs of DAP. The peptide-modified electrode has undergone the full procedure of detection as described in the experimental section (Target concentration, 0.32 μM). Particularly, a series of different incubation time for LH-catalyzed reaction has been adopted. (b) Peak currents in (a) plotted as the function of reaction time (Error bars mark the standard deviation, n=3).
